# Developmental, tract-tracing and immunohistochemical study of the peripheral olfactory system in a basal vertebrate: insights on Pax6 neurons migrating along the olfactory nerve

**DOI:** 10.1007/s00429-012-0486-2

**Published:** 2012-12-07

**Authors:** Idoia Quintana-Urzainqui, Isabel Rodríguez-Moldes, Eva Candal

**Affiliations:** Departamento de Biología Celular y Ecología, Edificio CIBUS Campus Vida, Universidad de Santiago de Compostela, 15782 Santiago de Compostela, Spain

**Keywords:** Migratory mass, Guidepost cells, Olfactory epithelium, Olfactory ensheathing glia, *Scyliorhinus canicula*, Dogfish

## Abstract

The olfactory system represents an excellent model for studying different aspects of the development of the nervous system ranging from neurogenesis to mechanisms of axon growth and guidance. Important findings in this field come from comparative studies. We have analyzed key events in the development of the olfactory system of the shark *Scyliorhinus canicula* by combining immunohistochemical and tract-tracing methods. We describe for the first time in a cartilaginous fish an early population of pioneer HuC/D-immunoreactive (ir) neurons that seemed to delaminate from the olfactory pit epithelium and migrate toward the telencephalon before the olfactory nerve was identifiable. A distinct, transient cell population, namely the migratory mass, courses later on in apposition to the developing olfactory nerve. It contains olfactory ensheathing glial (GFAP-ir) cells and HuC/D-ir neurons, some of which course toward an extrabulbar region. We also demonstrate that Pax6-ir cells coursing along the developing olfactory pathways in *S. canicula* are young migrating (HuC/D and DCX-ir) neurons of the migratory mass that do not form part of the terminal nerve pathway. Evidences that these Pax6 neurons originate in the olfactory epithelium are also reported. As Pax6 neurons in the olfactory epithelium show characteristics of olfactory receptor neurons, and migrating Pax6-ir neurons formed transient corridors along the course of olfactory axons at the entrance of the olfactory bulb, we propose that these neurons could play a role as guideposts for axons of olfactory receptor neurons growing toward the olfactory bulb.

## Introduction

The olfactory system is an excellent model for studying several developmental aspects of the nervous system, including cell migration, tract formation and guidance as well as adult neurogenesis. Its organization is well conserved in vertebrates and consists of the olfactory epithelium, which contains the olfactory receptor neurons (ORNs), the olfactory nerve composed of the axons from ORNs and supporting elements, and the olfactory bulb, the region of the telencephalon where olfactory axons make contact with central neurons, that is, the primary olfactory center.

The olfactory organ (where olfactory epithelium is placed) originates from a cephalic placode, the olfactory placode, which gives rise to olfactory and non-olfactory epithelia. Several studies have described the emergence of different cell populations from the olfactory placode and/or the olfactory epithelium and heading for telencephalic walls (Filogamo and Robecchi 1969; Schwanzel-Fukuda and Pfaff 1989; Wray et al. 1989; Drapkin and Silverman 1999; Schwanzel-Fukuda 1999; Puche and Baker 2007; Treloar et al. 2009; Blanchart et al. 2011). Although the placodal origin of these populations has been traditionally accepted, it is still a matter of discussion and the neural crest is considered as an alternative source for some of them (Whitlock 2004; Barraud et al. 2010; Forni et al. 2011; Katoh et al. 2011). Regardless of their origin, several cell populations have been described traveling along this pathway closely related to developing olfactory axons which seemed to serve as tracks for their migration (Mendoza et al. 1982; Farbman and Squinto 1985; Valverde et al. 1992, 1993; Pellier and Astic 1994; Pellier et al. 1994; De Carlos et al. 1996; Whitlock and Westerfield 1998; Fornaro et al. 2001, 2003; Balmer and LaMantia 2005; Maier and Gunhaga 2009; Miller et al. 2010; Blanchart et al. 2011). Some of these migrating cell populations are related to olfactory function. Among them, the olfactory ensheathing cells (OECs) represent a specific type of olfactory glia that ensheathes groups of unmyelinated olfactory axons and also plays an important role in guidance (Doucette 1984, 1990; Norgren et al. 1992; Chuah and West 2002; Franssen et al. 2007). This population exhibits antigenic and morphological characteristics of both astrocytes and Schwann cells and typically expresses the astrocyte-specific marker glial fibrillary acid protein (GFAP) (Barber and Lindsay 1982; Smithson and Kawaja 2009; Pellitteri et al. 2010; Forni et al. 2011). Neurons expressing olfactory receptor genes and the olfactory marker protein (OMP, a typical marker of some types of ORNs) have also been observed during development along the course of the olfactory nerve, where they have been proposed to act as “guidepost” cells for olfactory axons in their migration toward the olfactory bulb (Valverde et al. 1993; Conzelmann et al. 2002). Other migrating populations associated with the developing olfactory nerve do not appear directly related to olfactory functions. This is the case of a population apparently implicated in neocortical development (De Carlos et al. 1996), and the population of LHRH (luteinizing hormone-releasing hormone)/GnRH (gonadotropin-releasing hormone) expressing cells that are part of the terminal nerve system (Wray et al. 1989; Whitlock 2004).

Because of its key phylogenetic position as a representative species of the most ancient radiation of jawed vertebrates, the elasmobranch *Scyliorhinus canicula* (lesser spotted dogfish) has become a very suitable fish model in studies of vertebrate evolution and development (Coolen et al. 2009). Moreover, as other cartilaginous fishes, it possesses a highly developed sense of smell that is easily accessible to different experimental approaches, and represents an important emerging model for olfactory development studies. The structure and ultrastructure of the adult olfactory bulb and olfactory epithelium have been described in sharks and rays (elasmobranchs), including *S. canicula* (olfactory epithelium/placode: Theisen et al. 1986; Takami et al. 1994; Ferrando et al. 2006, 2007a, 2009, 2010; Schluessel et al. 2008; olfactory bulb: Dryer and Graziadei 1993, 1996) but, although some genetic data in early embryos are available (Sauka-Spengler et al. 2001; O’Neill et al. 2007) studies on the development of the olfactory system are really scarce (Fishelson and Baranes 1997; Ferrando et al. 2007b; Ferreiro-Galve et al. 2012a). As far as we know, no axonal tracing studies of the olfactory system have been performed in developing cartilaginous fishes so far.

Pax6 is a transcription factor conserved from invertebrates to vertebrates defined by the presence of two highly conserved DNA-binding motifs: a paired domain (PD) in its N-terminus and a paired-like homeodomain in the middle, which bind to distinct DNA consensus sequences. Pax6 acts in crucial developmental processes in the central nervous system, eyes, nose, pancreas, and pituitary gland (reviewed in Osumi et al. 2008). It is, indeed, a pleiotropic player in development as it participates in multiple aspects such as control of proliferation and cell fate (Marquardt et al. 2001; Simpson and Price 2002; Philips et al. 2005; Oron-Karni et al. 2008; Osumi et al. 2008), patterning and boundary formation (Haubst et al. 2004) and development of axonal pathways (Jones et al. 2002; Pratt and Price 2006; Nomura et al. 2007). Studies of loss of function and Pax6 mutants highlighted this gene as necessary for the normal development of the olfactory system (Hogan et al. 1986; Grindley et al. 1995; Anchan et al. 1997; Jiménez et al. 2000). The expression and possible functions of this transcription factor have been largely studied in the olfactory system of vertebrates, where it appears to be involved in the development of the olfactory placode and olfactory epithelium, the generation of specific interneuron subtypes in the postnatal olfactory bulb, positioning and axon guidance of neurons within the olfactory bulb and migration and alignment of olfactory cortex neurons (reviewed in Nomura et al. 2007). Furthermore, Pax6 expressing cells have been first described along the course of the olfactory nerve in *S. canicula* (Ferreiro-Galve et al. 2012a), although the nature of these cells and their relation to the olfactory nerve development was not determined. The characterization of the phenotype of these cells is crucial to ascertain the involvement of Pax6 in the development of the dogfish olfactory nerve.

In this study, we have analyzed the development of the olfactory system in the shark *S. canicula* with two aims: (1) to identify the early formation of the olfactory epithelium and primary olfactory projections in a cartilaginous fish, characterizing the different cell types of the olfactory epithelium and those associated with the developing olfactory nerve; and (2) to discern the phenotype of Pax6 cells at the olfactory epithelium and olfactory nerve to shed light on their possible role(s). To meet these goals, we have implemented tract-tracing techniques in combination with immunohistochemistry with markers for Pax6, proliferating cells (PCNA, proliferative cell nuclear antigen), glial cells (GFAP, glial fibrillary acidic protein), early postmitotic neurons (HuC/D), migrating immature neurons and their growing fibers (DCX, doublecortin) and mature ORNs and primary tracts (Gα_0_-protein). This study represents the first detailed analysis of the development of the olfactory system in an elasmobranch species and provides important information about the basic developmental processes that take place during the formation of olfactory structures in basal vertebrates; thus, helping to understand how the vertebrate olfactory system has evolved. Unraveling the details of the olfactory development in cartilaginous fish is crucial, not only for comparative purposes (as they are among the vertebrates with highly developed sense of smell), but also to shed light on how the relations between peripheral sense organs and central olfactory structures are established.

## Materials and methods

### Experimental animals

Some embryos of the lesser spotted dogfish (*Scyliorhinus canicula*) were supplied by the *Station Biologique* of Roscoff (France) and the *Estación de Bioloxía Mariña da Graña* (Galicia, Spain). Additional embryos were kindly provided by the Aquaria of Gijón (Asturias, Spain), O Grove (Pontevedra, Spain) and *Finisterrae* (A Coruña, Spain). Embryos were staged by their external features following Ballard et al. (1993). For more information about the relationships of the embryonic stages, body size, gestation and birth, see Table 1 in Ferreiro-Galve et al. (2010). Fifty-six embryos from stages 21 to 34 and five juvenile dogfish were used. Eggs from different broods and juveniles were raised in seawater tanks in standard conditions of temperature (15–16 °C), pH (7.5–8.5) and salinity (35 g/L). Adequate measures were taken to minimize animal pain or discomfort. All procedures conformed to the guidelines established by the European Communities Council Directive of 24 November 1986 (86/609/EEC) and by the Spanish Royal Decree 1201/2005 for animal experimentation and were approved by the Ethics Committee of the University of Santiago de Compostela.

### Tissue processing

Embryos were deeply anesthetized with 0.5 % tricaine methane sulfonate (MS-222; Sigma, St. Louis, MO) in seawater and separated from the yolk before fixation in 4 % paraformaldehyde (PFA) in elasmobranch’s phosphate buffer [EPB: 0.1 M phosphate buffer (PB) containing 670 mM urea, pH 7.4] for 48–72 h depending on the stage of development. Embryos from stage 32 onwards and juveniles were deeply anesthetized with MS-222 and then perfused intracardially with elasmobranch Ringer’s solution (see Ferreiro-Galve et al. 2012b) followed by 4 % PFA in EPB. The brains with the olfactory organs attached were removed and postfixed in the same fixative for 24–48 h at 4 °C. Subsequently, they were rinsed in phosphate buffered saline (PBS), cryoprotected with 30 % sucrose in PB, embedded in OCT compound (Tissue Tek, Torrance, CA), and frozen with liquid nitrogen-cooled isopentane. Parallel series of sections (18–20 μm thick) were obtained in sagittal, horizontal or transverse planes on a cryostat and mounted on to Superfrost Plus (Menzel-Glasser, Madison, WI, USA) slides.

### Immunohistochemistry

For heat-induced epitope retrieval, sections were pre-treated with 0.01 M citrate buffer (pH 6.0) for 30 min at 95 °C, and allowed to cool for 20–30 min at room temperature (RT). Sections were rinsed twice in 0.05 M Tris-buffered saline (TBS; pH 7.4) for 5 min each, and incubated overnight at RT with the following primary antibodies (see Table 1): mouse monoclonal anti-human HuC/HuD (HuC/D), rabbit and goat polyclonal anti-Pax6, rabbit and goat polyclonal anti-doublecortin (DCX), rabbit polyclonal anti-glial fibrillary acidic protein (GFAP), mouse monoclonal anti-proliferating cell nuclear antigen (PCNA) and rabbit polyclonal anti-Gα_0_-protein. Sections were rinsed twice in 0.05 M Tris-buffered saline (TBS) pH 7.4 for 5 min each, and incubated in the appropriate fluorescent dye-labeled secondary antibody (see Table 1) for 2 h at RT. All dilutions were made with TBS containing 15 % normal donkey serum (Millipore, Billerica, MA) 0.2 % Triton X-100 (Sigma) and 2 % bovine serum albumin (BSA, Sigma). All incubations were carried out in a humid chamber. Sections were then rinsed in TBS for 30 min and in distilled water (twice for 30 min). Sections were then allowed to dry for 2 h at 37 °C, and mounted in MOWIOL 4-88 Reagent (Calbiochem, MerkKGaA, Darmstadt, Germany). Additional information about the primary and secondary antibodies is included in Table 1.

**Table 1:** Primary and secondary antibodies

Primary antibody	Source	Working dilution	Secondary antibody	Source	Working dilution
HuC/D	Monoclonal mouse anti-HuC/D	1:100	546-conjugated donkey anti-rabbit (DAR^546^)	Molecular probes	1:100
				Catalog number: A10040	
	Molecular Probes, Eugene, OR				
	Catalog number: A-21271				
Pax6	Polyclonal rabbit anti-Pax6	1:200	546-conjugated donkey anti-mouse (DAM^546^)	Molecular probes	1:200
	Covance, Emeryville, CA			Catalog number: A10036	
	Catalog number: PRB-278P				
Pax6	Polyclonal goat anti-Pax6	1:100	488-conjugated donkey anti-rabbit (DAR^488^)	Molecular probes	1:100
	Novus Biologicals, Littleton, CO			Catalog number: A21206	
	Catalog number: NB100-2913				
DCX	Polyclonal rabbit anti-DCX	1:300	488-conjugated donkey anti-mouse (DAM^488^)	Molecular probes	1:100
	Cell Signaling Technology, Beverly, MA.			Catalog number: A21202	
	Catalog number: 4604				
DCX	Polyclonal goat anti-DCX	1:100	488-conjugated donkey anti-goat (DAG^488^)	Molecular probes	1:100
	Santa Cruz Biotechnology, Santa Cruz, CA			Catalog number: A11055	
	Catalog number: sc-8066				
GFAP	Polyclonal rabbit anti-GFAP	1:500	633-conjugated donkey anti-goat (DAG^633^)	Molecular probes	1:150
	Dako, Glostrup, Denmark			Catalog number: A21082	
	Catalog number: Z 0334				
PCNA	Monoclonal mouse anti-PCNA	1:300	633-conjutated donkey anti-mouse (DAM^647^)	Molecular probes	1:100
	Sigma, St. Louis, MO			Catalog number: A31571	
	Catalog number: P8825				
Gα_0_	Polyclonal rabbit anti-Gα_0_	1:400			
	Santa Cruz Biotechnology, Santa Cruz, CA				
	Catalog number: sc-387				

### Double and triple immunofluorescence

Double and triple immunolabeling were performed on alternate series of sections, which were incubated overnight at RT with cocktails of primary antibodies mixed at optimal dilutions and subsequently detected using mixtures of appropriate secondary antibodies. All incubations were carried out in a humid chamber and processed as described above. Double immunofluorescence with primary antibodies raised in the same species was performed as described in Tornehave et al. (2000).

### Controls and specificity of the antibodies

Control experiments were carried out by omitting the primary or secondary antiserum in the incubations. No immunostaining was detected in any case.

Mouse monoclonal anti-HuC/D has been shown to specifically label neuronal cells in zebrafish, birds and humans (Marusich et al. 1994; Ekström and Johansson 2003; Soukkarieh et al. 2007; Vellema et al. 2010). The same antibody labeled different types of neurons in the spinal cord of embryos and juveniles of the lesser spotted dogfish (Sueiro et al. 2004).

The rabbit polyclonal and the goat polyclonal Pax6 antibodies were raised against peptides derived from the C-terminus of the mouse and human peptide, respectively. Multiple sequence alignment (Corpet 1988) of Pax6 (GenBank NP_000271.1), Pax6-5a (GenBank NP_001595.2), and Pax6-∆PD (GenBank AAL40860) showed that the C-terminus of the protein recognized by the rabbit antibody is identical in the three Pax6 isoforms (Ferreiro-Galve et al. 2012b). The pattern of immunostaining was identical using both antibodies. The specificity of the immunoreaction of both Pax6 antibodies in the retina and brain of *S. canicula* was previously tested in our laboratory by pre-adsorbing the primary antibodies with the antigenic Pax6 peptide (NB100-2913PEP; Novus Biologicals, Littleton, CO) used for generation of the NB100-2913 antiserum. For both antibodies, the immunostaining was completely abolished in sections treated with primary antibodies at working dilution and preadsorbed with the blocking peptide (see Ferreiro-Galve et al. 2012b).

The rabbit polyclonal and goat polyclonal anti-DCX antibodies recognized a single band of approximately 45 kDa (manufacturer’s technical information). In our hands, the results obtained with both antibodies were identical. We tested the specificity of the immunoreaction for both DCX antibodies in the brain of *S. canicula* by pre-adsorbing the primary antibodies with the antigenic DCX peptide (sc-8066P; Santa Cruz Biotechnology, Santa Cruz, CA). The immunostaining was completely abolished in *S. canicula* sections treated with primary antibodies at working dilution after pre-adsorption with the blocking peptide at a concentration of 10 mg/mL at 4 °C for 24 h.

The polyclonal anti-GFAP antibody is a purified immunoglobulin fraction of rabbit antiserum generated to bovine spinal cord GFAP. This antibody has been previously used as an immunohistochemical marker for olfactory ensheathing cells (Katoh et al. 2011) and as a glial marker in *S. caninula* (Wasowicz et al. 1999; Sueiro et al. 2007).

The monoclonal anti-PCNA recognizes a protein of 36 kDa corresponding to the acidic non-histone auxiliary protein of DNA polymerase, according to the manufacturer. This antibody specifically labels proliferating cells in the brain and olfactory epithelium of *S. canicula* (Rodríguez-Moldes et al. 2008; Ferrando et al. 2010).

The Gα_0_ (K-20) antibody is an affinity purified rabbit polyclonal antibody raised against a peptide mapping within a highly divergent domain of the trimeric G protein subunit Gα_0_ of rat origin. In Western blots of protein homogenates of the olfactory organ of *S. canicula*, this antiserum recognizes a single band of approximately 42 kDa (Ferrando et al. 2009). In *S. canicula*, this antibody has been previously used to label olfactory receptor neurons and axon bundles in the *fila olfactoria* (Ferrando et al. 2009).

### In vitro tract-tracing techniques

A total number of 10 animals ranging from stage 28-embryos to juveniles were used. The experiments were performed under in vitro whole-brain conditions. All specimens were deeply anaesthetized in seawater containing 0.5 % MS-222. Animals were then immersed in (stages 28 and 31) or perfused transcardially with (stage 32 onwards) 30 mL of ice-cooled elasmobranch Ringer’s solution containing 1 mM glucose, which was oxygenated with an oxygen injector aerator to a pH of 7.4. After the animal decapitation, the brains were isolated by removing the overlying cartilage skull and skin and transferred to fresh Ringer’s solution to proceed with the immediate application of the tracer. We applied neurobiotin (Vector Laboratories, Burlingame, CA), an amino derivative of biotin used as an intracellular label for neurons, whose transport efficiency in retrograde labeling has been proved (Barreiro-Iglesias et al. 2008). The tracer was dissolved in distilled water until saturation and re-crystallized at the tip of an entomological needle (00) according to Morona et al. (2005) and the application was carried out manually or using a micromanipulator (Narishige MN-151, Japan) under a stereomicroscope. The olfactory epithelium and the olfactory bulb were accessed by vertical penetrations and all applications were made unilaterally. After tracer application, brains were maintained for 2–3 days at 8 °C in continuously oxygenated elasmobranch Ringer’s solution containing 1 mM glucose, and then fixed for 2 days in 4 % paraformaldehyde in EPB. The tissue was cryoprotected and sectioned as explained above. Neurobiotin was visualized by incubating the sections with fluorescein isothiocyanate (FITC)-labeled avidin D (Vector Laboratories; diluted 1:1,000 in PBS containing 0.2 % Triton X-100) in a humid chamber for 2.5 h at 37 °C. The slides were rinsed in TBS, then in distilled water, dried for 30 min at 37 °C, and mounted in MOWIOL.

### Combined tract-tracing and immunohistochemistry

For combined tract-tracing and immunohistochemistry, the tracer was applied and the tissue was processed as described above. Primary antibody solutions and avidin B were simultaneously incubated and revealed and mounted as previously explained.

### Bisbenzimide staining

Fluorescent counterstaining of cell nuclei was carried out by immersing the samples in 0.5 μg/ml bisbenzimide (Sigma) in TBS for 5 s. The labeling was visualized with an UV filter coupled to a spectral confocal laser scanning microscope TCS-SP2 (Leica, Wetzlar, Germany).

### Apoptosis detection

We assessed cell death in the olfactory system of *S. canicula* with the TUNEL technique using the NeuroTACS II in situ apoptosis detection kit (Trevigen, Inc., Gaithersburg, MD; catalog number 4823-30-K). Sections were treated according to the manufacturer to detect DNA fragments generated by apoptosis using a highly purified terminal deoxynucleotidyl transferase enzyme (TdT) and to reveal the incorporated nucleotides using a horseradish peroxidase system. Positive and negative controls were performed. For positive control, sections treated with TACS-Nuclease™ (Trevigen) to generate DNA breaks in every cell showed pale staining in most of cells after the application of the apoptosis detection kit. No labeling was observed when TdT was not included in the reaction.

#### Image acquisition and analysis

Double-labeled sections were analyzed and photographed with the TCS-SP2 scanning microscope with a combination of blue and green excitation lasers. Stacks of confocal images were acquired separately for each laser channel with steps of 0.8 or 2 μm along the *z*-axis and collapsed images were obtained from an average of 12 optical sections with the LITE software (Leica). Some sections were photographed with an epifluorescence photomicroscope Olympus AX70 fitted with an Olympus DP70 color digital camera. Sections of apoptosis experiments were photographed with an Olympus BX51 microscope equipped with an Olympus DP71 color digital camera. Photographs were adjusted for brightness and contrast and plates were prepared using Adobe Photoshop CS4 (Adobe, San Jose, CA). Images were not otherwise modified.

## Results

**Fig. 1:**
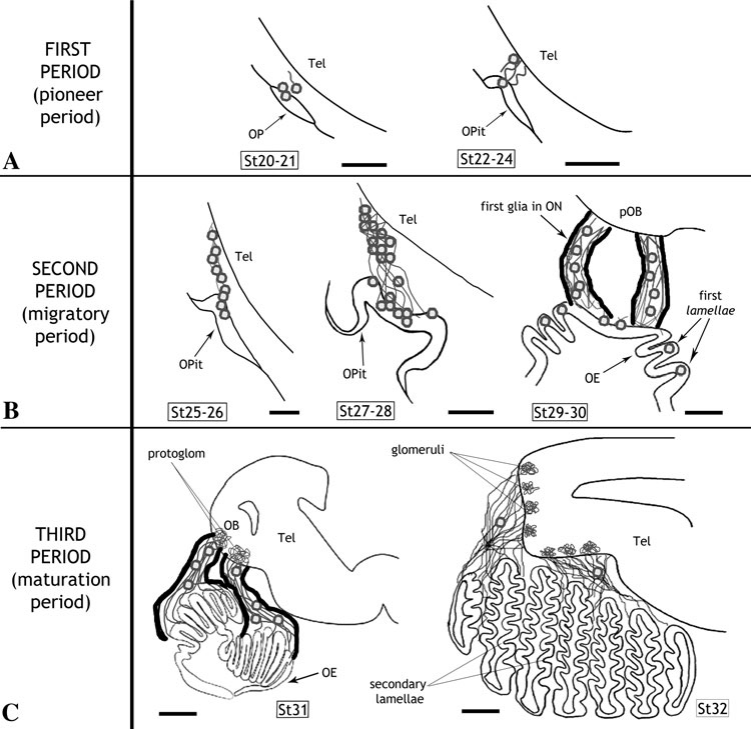
Schematic drawings representing the main key events of the development of peripheral components of the lesser spotted dogfish olfactory system. *Dots* represent immature postmitotic neurons (and respective projections represented as *grey lines*). *Thick black lines* in **b**, **c** represent glial processes surrounding the olfactory nerve. For abbreviations, see list. *Scale bars* 100 μm (**a**), 150 μm (**b**), and 250 μm (**c**)

We have identified the main key events that take place during the development of the olfactory system in the lesser spotted dogfish (*S. canicula*) and we have framed them into the context of the three developmental periods that were established on the basis of tract-tracing experiments and the expression of various immunohistochemical markers (see Fig. 1; Table 2). These events and developmental periods constitute a useful reference for comparative developmental studies of the olfactory system as they can be consistently identified in different vertebrates (see “Discussion”). The first period (Fig. 1a, stages 20–24) is marked by the onset of neurogenesis and the observation of the first postmitotic neurons in the olfactory placode, which seem to pioneer the olfactory pathway across the mesenchyme. The second period (Fig. 1b, stages 25–30) is characterized by profuse neuronal migration along the olfactory fibers. During the third period (Fig. 1c, stage 31 to hatching), the mature structure of the olfactory system components is progressively acquired. Consequently, we have termed these periods pioneer, migratory and maturation periods, respectively.

**Table 2:** Key events during the development of the peripheral components of *S. canicula* olfactory system

	First period St20–St24 (Pioneer period)	Second period St25–St30 (Migratory period)	Third period St31–St34/prehatching (Maturation period)
OE	Formation of the OP First neuronal precursors (establishment of neurogenic region) Indentation of the OP Formation of the OPit	Invagination of the OPit Expansion of the neurogenic region (St29) First folds (primary *lamellae*)	Stratification Secondary *lamellae* Mature morphology (St32)
ON	Pioneering axons and neurons	Massive neuronal migrations along ON First evidence of glia ensheathing ON(St29)	Increase in thickness of nerve fascicles Gradual disappearance of migrating neurons

### First period: pioneer period (stages 20–24)

**Fig. 2:**
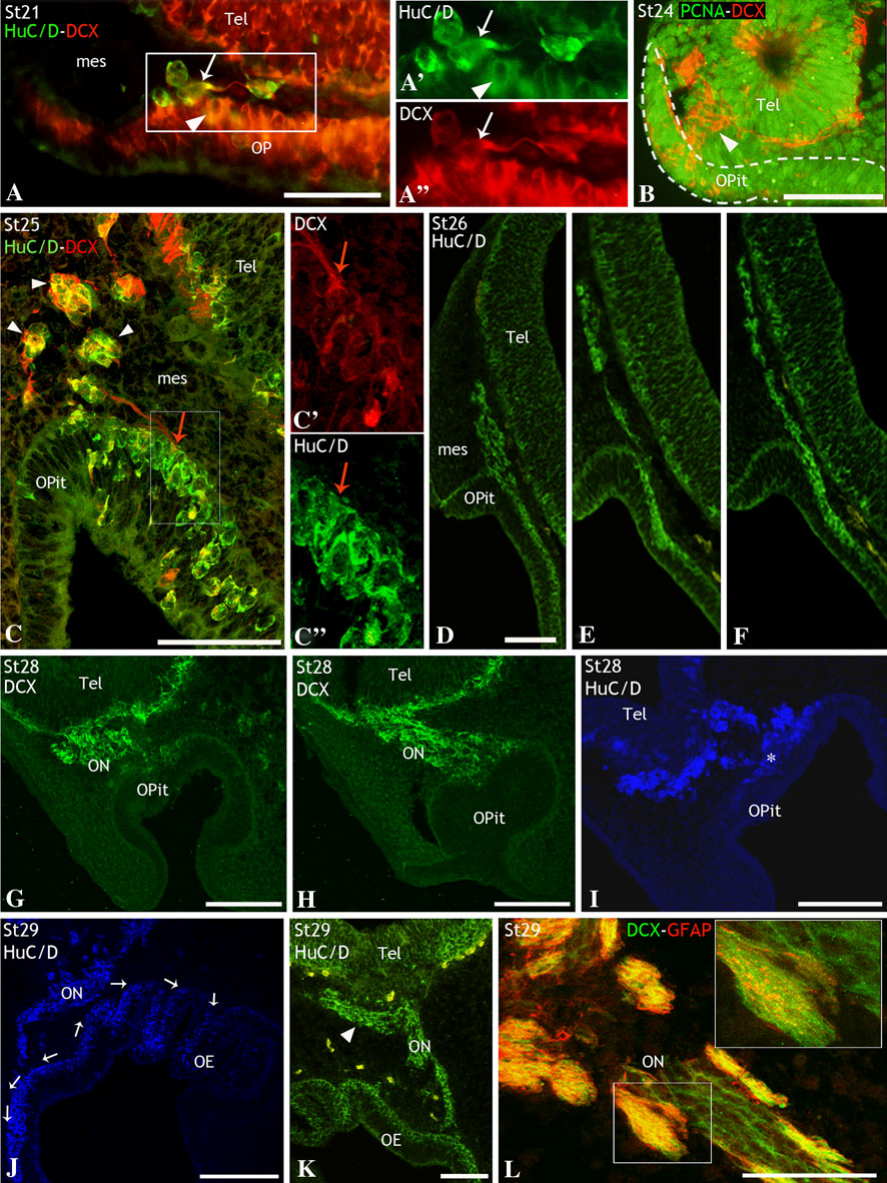
Sagittal sections of embryos at stages 21 and 24 (first period, **a**, **b**) and 25, 26, 28 and 29 (second period, **c**–**l**) showing the early development of the olfactory placode, olfactory pit and olfactory nerve. **a**, **a**″ Detail of the olfactory placode with immature postmitotic HuC/D-ir neurons (*arrowhead*), some of them delaminated from the olfactory placode and extending short DCX-ir processes across the mesenchyma (*arrow*). **b** Lateral section of the olfactory pit of a stage-24 embryo, where most cells showed PCNA immunoreactivity. Note also DCX-ir fibers emerging from cells located within the epithelium (*dotted line*) and extending to the telencephalon (*arrowhead*), probably establishing their first contacts. **c**–**c**″ Section of the olfactory pit to show the neurogenic region defined by its HuC/D expression. Some double HuC/D- and DCX-ir cells (*arrowheads*) were observed associated with DCX-ir fibers from neurons anchored to the epithelium (*red arrow*). **d**–**f** Sequence of consecutive sections showing a string of HuC/D-ir cells extending across the mesenchyme between the olfactory pit and the telencephalon. **g**–**i** Adjacent sections of the olfactory epithelium of a stage-28 embryo to show the increased number of DCX-ir fibers (**g**, **h**) and HuC/D-ir neurons (**i**) along the developing olfactory nerve with respect to previous stages. **g**, **i** Correspond to medial sections throughout the epithelium and **h** corresponds to a lateral section. *Asterisk* in **i** shows the basal position of HuC/D-ir postmitotic immature neurons within the olfactory epithelium. **j**–**l** Sections of the olfactory epithelium and olfactory nerve of stage-29 embryos to show the epithelium organized in primary lamellae and the medial to lateral expansion of the neurogenic region within the olfactory epithelium (in **j**, *arrows* indicate the direction of the expansion); a cluster of HuC/D-ir cells at the level of the olfactory nerve–olfactory bulb junction that branched off from the olfactory pathway (*arrowhead*) towards more anterior telencephalic levels (**k**); and GFAP-ir glial processes that surrounded the DCX-ir fibers of the olfactory nerve and strings of DCX-ir somata identified as immature migrating neurons (**l**). The *inset* in **l** is a detail of a single confocal section (1 μm thick) to show that there was no colocalization between both markers. For abbreviations, see list. *Scale bars* 50 μm (**a**); 100 μm (**b**, **c**, **k**, **l**); 150 μm (**d**–**j**)

The olfactory placode derives from the ectoderm on the ventrolateral surface of the head of the early embryo and becomes morphologically visible at stage 20 (Sauka-Spengler et al. 2001), which corresponds to the beginning of the first period. At this very early stage, most placodal cells showed PCNA immunoreactivity, but immunolabeling for markers of differentiation was not observed (data not shown). Shortly after (stage 21) the olfactory placode was visibly thickened and a few early postmitotic neurons were already detected by means of HuC/D immunoreactivity (arrowhead in Fig. 2a, a′). Some of the earliest HuC/D-immunoreactive (ir) neurons seemed to delaminate from the inner region of the placodal epithelium, extending short processes in the mesenchyme (arrow in Fig. 2a, a′). The earliest delaminated neurons also showed immunoreactivity to DCX, a marker for migrating immature neurons (Fig. 2a, a″). Because of their early emergence from the olfactory placode epithelium, we identified these cells as pioneer neurons, a transient class of neurons that prefigure the primary olfactory pathway before the expression of olfactory receptor genes and the outgrowth of olfactory axons (Whitlock and Westerfield 1998). Later in development (stage 22), the first signs of invagination of the olfactory placode took place. At this stage, DCX-ir axons of first pioneer neurons contacted with the surface of the telencephalon (not shown). During stages 22–24, the thickened placodal epithelium invaginated to form the olfactory pit (Fig. 1a). Most cells throughout the olfactory pit neuroepithelium continued showing PCNA immunoreactivity (Fig. 2b) although some postmitotic immature neurons (DCX-ir) whose axons reached the surface of the telencephalon were also observed (arrowhead in Fig. 2b). These axons of olfactory pit cells contacting the telencephalon represent the first evidence of the olfactory nerve primordium.

### Second period: migratory period (stages 25–30)

At stage 25 (Figs. 1b, 2c), the number of early postmitotic (HuC/D-ir) neurons of the olfactory pit epithelium increased. They were mainly located in a neurogenic region found in the medial/deeper zone of the developing olfactory epithelium where HuC/D-ir cells mostly occupied its basal part, close to the mesenchyme. At this stage, strongly DCX-ir fibers belonging to HuC/D-ir cells were observed exiting the neurogenic region (red arrows in Fig. 2c, c′, c″) and contributing to the primordial olfactory nerve. Concurrently, new subsets of HuC/D-ir and DCX-ir cells delaminated from this region of the olfactory pit and entered the adjacent mesenchyme in apposition to the developing olfactory nerve (arrowheads in Fig. 2c). Shortly after (stage 26), a stream of neurons was observed extending from the neurogenic region of the olfactory pit toward the surface of the telencephalon (Fig. 2d–f). At stage 28 (Figs. 1b, 2g–i), the invagination of the olfactory pit continued and the primordial olfactory epithelium expanded laterally to form a sac divided in two recesses with a common narrow entrance. As a result of these movements, the neurogenic region became situated in the medial part of the olfactory pit. The spatial extension of the neurogenic region within the olfactory pit was concurrent with an increasing number of postmitotic HuC/D-ir and DCX-ir immature neurons (Fig. 2g–i), which remained located in the basal part of the neuroepithelium (asterisk in Fig. 2i). There was also a marked increase in the number of olfactory fibers in the olfactory nerve, and of neurons migrating along it as revealed by their DCX (Fig. 2g, h) and HuC/D immunoreactivity (Fig. 2i), respectively. At stage 29, the olfactory epithelium began to fold forming the first characteristic lamellae (Figs. 1b, 2j, k) and the neurogenic region of the epithelium extended from medial to lateral aspects of the organ, as revealed the distribution of HuC/D-ir cells (see direction of arrows in Fig. 2j). At this stage, the primordial olfactory bulbs (pOB in Fig. 1b) became recognizable as small protrusions that emerged at the lateral walls of the telencephalon. Of note is the observation of a cluster of HuC/D-ir cells near the olfactory nerve-olfactory bulb contact region, which deviates from the olfactory pathway toward more anterior regions near the telencephalic wall (arrowhead in Fig. 2k). Although more studies are necessary to demonstrate it, we are persuaded that this branched population form part of the developing terminal nerve and ganglia. Also at this stage, the first evidence of the presence of olfactory glia in the olfactory nerve was obtained by means of GFAP immunohistochemistry (Figs. 1b, 2l). Glial processes (GFAP-ir) were arranged surrounding bundles of DCX-ir cells, characterized as migrating immature neurons, and DCX-ir fibers (Fig. 2l). Colocalization of GFAP and DCX in the same processes was ruled out after analyzing 1-μm confocal sections (see inset in Fig. 2l).

### Third period: maturation period (stages 31–34/prehatching)

**Fig. 3:**
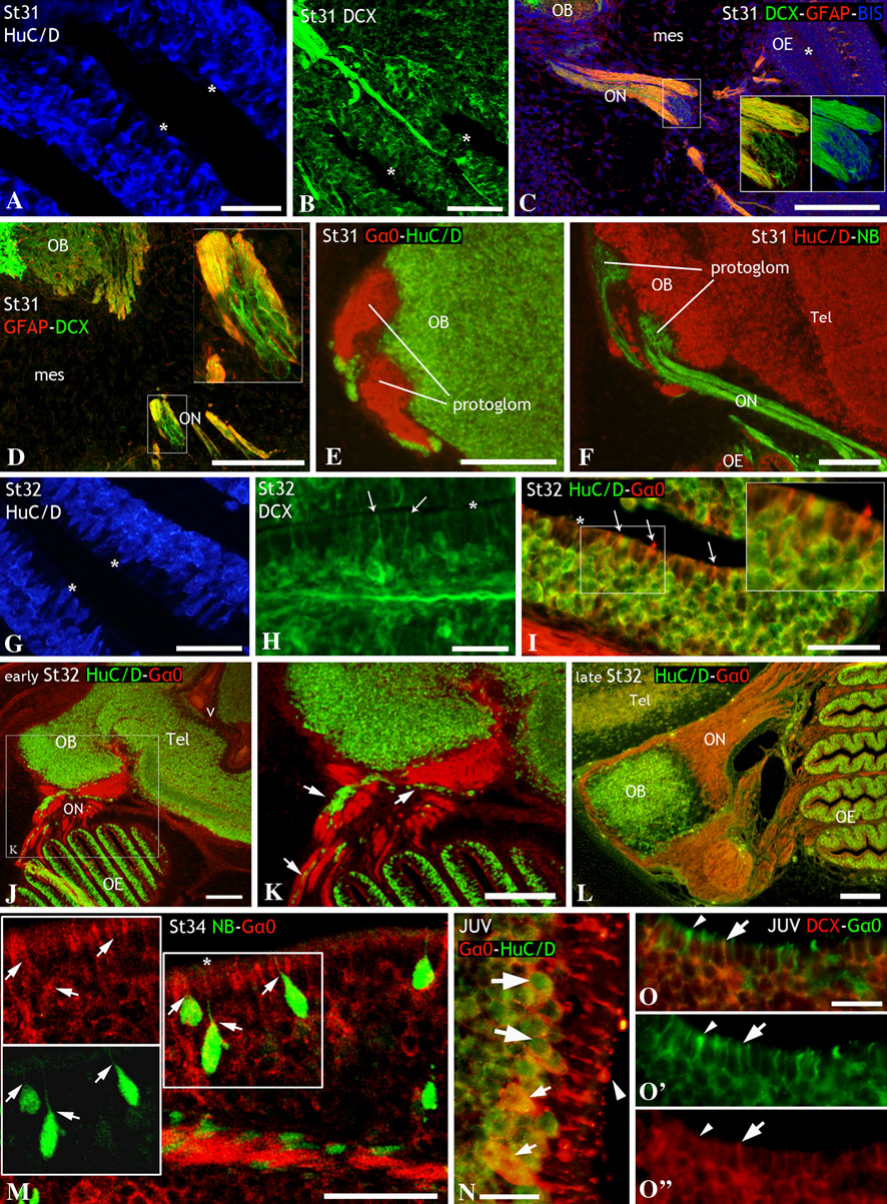
Sections of embryos at stages 31–34 (third period, **a**–**l**), and of juveniles (**m**–**o**) to show the late development of the olfactory epithelium and olfactory nerve, and the early development of the olfactory bulb. **a**, **b** Details of lamellae of the olfactory epithelium at stage 31 to show the scattered distribution of HuC/D-ir (**a**) and DCX-ir (**b**) neurons. Note in **b**, fascicles of DCX-ir fibers exiting the olfactory epithelium. **c**, **d** Double DCX/GFAP immunofluorescence in sections across the olfactory nerve at stage 31 to show the ensheathing GFAP-ir glial fibers around DCX-ir migrating neurons and fibers. Bisbenzimide counterstaining of cell nuclei is showed in **c**. *Insets* in **c** and **d** are details to show the absence of colocalization between DCX and GFAP. **e**, **f** Transverse sections of the telencephalon at stage 31 to show the first evidence of protoglomerular fields, revealed by Gα_0_ immunohistochemistry (**e**) and tract-tracing experiments (**f**). Note the absence of HuC/D-ir neurons within protoglomerular fields and the presence of Gα_0_ immunoreactive and anterogradely labeled fibers, respectively. **g**–**i** Sections through olfactory lamellae at stage 32 to show the stratification of cells in the epithelium. Note that HuC/D-ir and DCX-ir perikarya were absent from apical layer (**g**, **i**) and that some DCX-ir cells (*arrows* in **h**) presented a bipolar morphology with a dendrite directed towards the apical surface of the epithelium, indicated with *asterisk*. The *inset* in *i* is a detail to show that the dendrites of most neurons contain the ORN marker Gα_0_ (*arrows*). **j**–**l** Transverse sections to comparatively show the decreasing density of HuC/D-ir neurons (*white arrows*) along olfactory nerves as the development of stage-32 embryos proceeds. **k** Is a detail of the area *squared* in **j**. The primary olfactory projection was revealed by its immunoreactivity to the Gα_0_-protein. **m** Transverse sections of the olfactory epithelium of a prehatching embryo showing cells immunoreactive to the Gα_0_-protein (ORNs) that were retrogradely labeled after application of neurobiotin in the olfactory bulb (*arrows*). **n**, **o** Transverse sections through the olfactory epithelium of juveniles showing the intense Gα_0_ immunolabeling of mature ORNs (*small arrows*), specially in the apical dendrite (*arrowhead* in **n**, **o**, **o**′). *Arrowhead* in **o**″ points to the DCX immunonegative dendrite of a mature ORN (intensely Gα_0_-ir). *Large arrows* in **o**–**o**″ indicate a maturing ORN that shows weak levels of both DCX and Gα_0_-protein. *Asterisks* in **a**–**c**, **g**–**i**, and **m** indicate the apical surface of the olfactory epithelium. For abbreviations, see list. *Scale bars* 75 μm (**a**); 50 μm (**b**, **g**); 300 μm (**c**); 150 μm (**d**); 200 μm (**e**, **f**, **j**, **k**, **l**); 25 μm (**h**, **m**, **o**); 100 μm (**i**); 10 μm (**n**)

At stage 31, the olfactory epithelium became further folded (Fig. 1c) and neurogenesis extended to the entire epithelium. Cells immunoreactive to HuC/D (Fig. 3a) and DCX (Fig. 3b) appeared throughout the epithelium, but the scattered distribution of these markers indicates that the stratification of neurons in the epithelium has not yet occurred. The structure of the olfactory nerve was similar to that observed in previous stages, i.e., bundles of DCX-ir cells (migrating neurons) and fibers appeared surrounded by glial processes (GFAP-ir) (Fig. 3c, d), although the nerve fascicles were thicker than in previous developmental stages. In the olfactory bulb, first protoglomeruli were noticed by their intense immunoreactivity to the Gα_0_-protein (Fig. 3e). The whole primary projection at this stage was evidenced by massive application of neurobiotin to the olfactory epithelium (Fig. 3f). In these experiments, the olfactory bundles ended in two rather compact protoglomerular fields surrounded by HuC/D neurons of the bulb.

As development proceeded (stage 32), secondary epithelial lamellae extended perpendicular to the primary *laminae* (Fig. 1c) and stratification of olfactory neurons through the height of the epithelium became evident as shown with different neuronal markers (Fig. 3g, h). Proliferating (PCNA-ir) cells were found throughout the olfactory epithelium, although PCNA immunoreactivity was more intense at the basal part (not shown). Cells immunoreactive to HuC/D (Fig. 3g) and DCX (Fig. 3h) occupied most of the epithelium, except the apical layer that eventually will contain sustentacular (non-neuronal) cells. Some DCX-ir cells showed bipolar morphology with an apical dendrite that reached the apical surface (arrows in Fig. 3h). The same morphology was observed in cells that showed immunoreactivity to Gα_0_-protein, especially in the apical dendrite ending in an olfactory knob (arrowheads in Fig. 3i) and in those that we have identified as the first maturing ORNs. At this stage, the density of HuC/D-ir cells along olfactory fibers was notably reduced in relation to previous stages (Fig. 3j, k) and they disappeared at the end of this stage (Fig. 3l). As development proceeded, there was an increase in the number of mature ORNs (mORNs), as revealed by their intense Gα_0_-protein immunoreactivity. Similar bipolar neurons were retrogradely labeled after massive application of neurobiotin in the olfactory bulb (Fig. 3m). These ORNs showed apical dendrites of variable lengths depending of the location of the perikaryon in the epithelium. The density of mORNs showing strong Gα_0_ immunoreactivity increased in postnatal specimens with respect to embryos (Fig. 3n, o). At juvenile stages, Gα_0_-ir cells that showed immunoreactivity to HuC/D and DCX were relatively abundant (large arrows in Fig. 3n, o). Of note, DCX immunoreactivity was observed in the apical processes of maturing (young) ORNs that showed low expression for Gα_0_-protein (large arrow in Fig. 3o, o′, o″), while it was absent in mature ORNs that expressed high levels of Gα_0_-protein (arrowhead in Fig. 3o, o′, o″).

### Characterization of Pax6 cell populations in the peripheral olfactory system

The presence and distribution of Pax6-expressing cells in developing and adult olfactory system of the lesser-spotted dogfish has been recently reported by some of us (Ferreiro-Galve et al. 2012a), who presented the first evidence in vertebrates of strings of Pax6-expressing cells extending along the developing olfactory nerve. In the present study, we have not only confirmed the existence of such cells and extended the search into earlier developmental stages to shed light on its origin but more importantly, we have characterized their nature through different developmental stages.

**Fig. 4:**
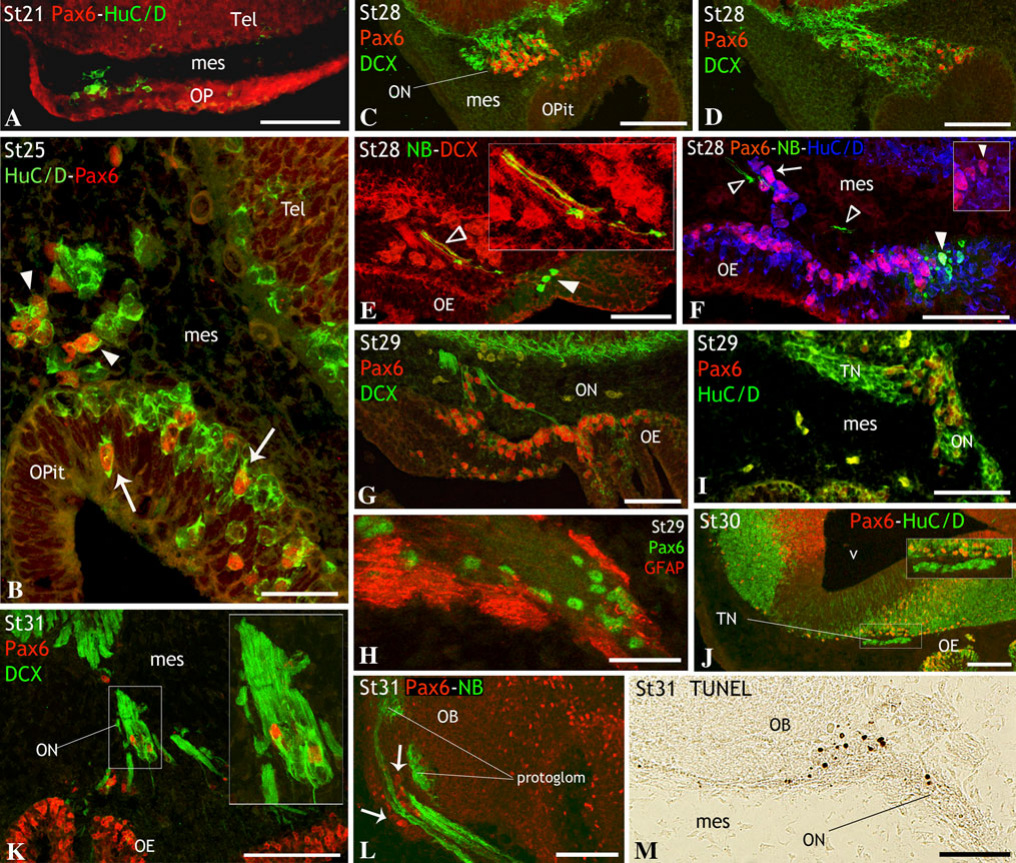
Pax6 expression in relation to the components of the olfactory system during development. Sagittal sections through the head of embryos at stages 21 (first period, **a**), 25–30 (second period, **b**–**j**) and 31 (third period) (**k**–**m**). **a** In stage embryos, Pax6 expression was observed throughout the olfactory placode, being faint in the neurogenic region from which HuC/D-ir pioneer cells delaminated. **b** Section of the olfactory pit at stage 25 to show high levels of Pax6 expression in a subset of HuC/D-ir neurons in the neurogenic region (*arrows*) and the mesenchyme (*arrowheads*). This section is parallel to that of Fig. 2c. **c**, **d** Adjacent sections of the olfactory epithelium of a stage-28 embryo to show high numbers of Pax6-ir cells neighboring DCX-ir fibers that invaded the mesenchyme. **e**, **f** Adjacent sections of a stage-28 embryo after application of neurobiotin into the olfactory epithelium, to show anterogradely labeled cells (*arrowheads*) and their outgrowing axons (*open arrowheads*). *Inset* in **e**: detail to show that neurobiotin-labeled fibers were DCX immunoreactive. The *inset* in **f** shows that some of these neurobiotin-labeled cells (maturing ORNs) were Pax6-ir (*arrowhead*). Note in **f** Pax6/HuC/D-ir cells (immature neurons) along unlabeled olfactory axons (*arrow*). **g** Section of the olfactory epithelium of a stage-29 embryo to show the increased number of Pax6-ir cells within the olfactory epithelium and some Pax6-ir cells in apposition to the DCX-ir olfactory fibers. **h** Detail of the olfactory nerve of a stage-29 embryo double labeled for GFAP and Pax6 to show that there were no colocalization between these markers. **i** Section across the olfactory nerve-olfactory bulb junction at stage 29 (compare with Fig. 2k). Pax6 (HuC/D-ir) neurons seemed to accumulate at this point as they never were detected along the terminal primordium. **j** Section of the head showing the string of terminal HuC/D-ir cells apposed to the ventral part of the telencephalic hemisphere. *Inset*: detail of the *squared* area to show Pax6 immunonegativity of primordial terminal ganglion cells. **k** Section of a stage-31 embryo to show the increased number of Pax6-ir cells within the olfactory epithelium and the scarce number of Pax6-ir cells within the olfactory nerve, identified by the DCX immunoreactivity, as detailed in the *inset*. **l** Section of the olfactory bulb of a stage-31 embryo to show that Pax6-ir cells formed corridors along the entrance of bundles of neurobiotin-labeled olfactory axons in the olfactory bulb (*arrows*). **m** Section of the olfactory bulb of a stage-31 embryo to show apoptotic (TUNEL positive) cells at the entrance of the olfactory bulb, which mirrored the position of Pax6 cells at the same stage. *Scale bars* 100 μm (**a**, **i**, **m**); 150 μm (**c**, **d**, **k**, **l**); 50 μm (**b**, **h**); 75 μm (**e**–**g**, **j**)

At stage 21, Pax6 was expressed throughout the olfactory placode but not in cells adjacent to the placode (Fig. 4a), which showed HuC/D immunoreactivity and appeared to delaminate from the neurogenic region (see above, first period). The expression of Pax6 immunoreactivity in this neurogenic region appeared to be lower than in the surrounding ectoderm (Fig. 4a). Conversely, during the second period (Fig. 4b–i) Pax6 immunoreactivity was intense in a subset of cells located in the neurogenic area of the olfactory epithelium (arrows in Fig. 4b), as well as in a subset of migrated cells (arrowheads in Fig. 4b) that were in apposition to the DCX-ir fibers of the developing olfactory nerve (see also Fig. 4c, d).

To assess the nature of these Pax6-ir cells, we applied neurobiotin to the olfactory epithelium of stage 28 embryos, which allowed the labeling of a few developing olfactory neurons per specimen and their respective axons (Fig. 4e). Experiments combining tract-tracing and DCX immunohistochemistry showed that neurobiotin-labeled axons were also immunoreactive to DCX (Fig. 4e). Other experiments with different immunomarkers revealed that some neurobiotin-labeled cells in the olfactory epithelium were double Pax6-ir/HuC/D-ir neurons (arrowhead and detail in Fig. 4f) that extended their axons toward the telencephalon (open arrowheads in Fig. 4f). Moreover, we observed migrated Pax6-ir neurons associated with these neurobiotin-labeled axons that did not contain the tracer (arrow in Fig. 4f), which suggests that they were not contacting with the olfactory epithelium surface at the moment of the tracer application. The density of Pax6-ir cells closely associated with the DCX-ir axons of the olfactory nerve continued being high at stage 29 (Fig. 4g), but decreased from this stage onwards. As reported above, glial cells showing GFAP immunoreactivity were firstly detected at this stage 29 and, as expected, colocalization between Pax6 and this glial marker was not observed (Fig. 4h), which ruled out a glial nature for the Pax6-ir cells associated with the olfactory nerve. Interestingly, while numerous Pax6-ir neurons were observed along the olfactory nerve, the number of these cells did not increase in the periphery of the olfactory bulb in a perceptible way. We considered the possibility that Pax6-ir cells deviate towards the terminal nerve pathway or, alternatively, they downregulate Pax6 expression or undergone programmed cell death at the entrance of the olfactory bulb. We observed that Pax6-ir neurons associated with the olfactory nerve seemed to accumulate distally in the olfactory path, close to the olfactory nerve–olfactory bulb junction (Fig. 4i) and they were absent along which could represent the terminal nerve pathway (Fig. 4i, j).

At the beginning of the maturation period (stages 31–34), a notable increase in the density of Pax6-ir cells was observed throughout the olfactory epithelium (Fig. 4k), but the density of Pax6-ir cells associated with the olfactory nerve notably diminished, as compared to previous developmental stages (Fig. 4k). Of note, Pax6-ir cells in the periphery of the olfactory bulb appeared to form corridors between the bundles of olfactory axons (see arrows in Fig. 4l). Interestingly, a number of apoptotic cells were detected at this stage at the entrance of the olfactory bulb (Fig. 4m), but not along the course of the olfactory nerve.

**Fig. 5:**
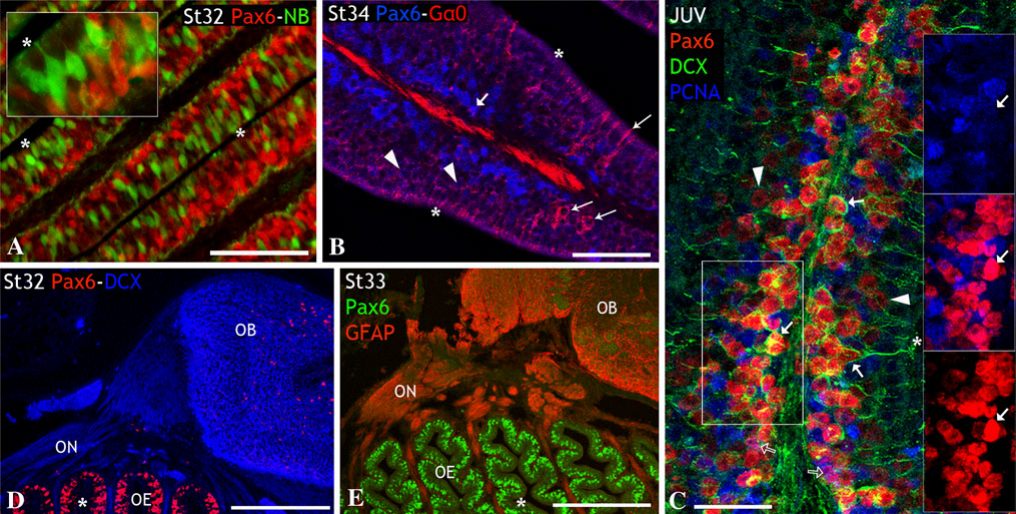
Pax6 expression during late third period (stages 32–34) (**a**, **b**, **d**, **e**) and juvenile (**c**). **a**–**c** Sections of the olfactory epithelium to show the changes in distribution of Pax6-ir cells as the epithelium became stratified. Note that Pax6 cells progressively adopted a more basal position as development proceeded (compare **a** and **b**). The *inset* in **a** shows that Pax6 immunoreactivity was absent from retrogradely labeled cells (mature ORNs) after application of neurobiotin to the olfactory bulb. Mature ORNs were also identified by its intense Gα_0_ immunoreactivity (*large arrows* in **b**; note also the absence of Pax6 expression in these cells). *Arrowheads* in **b** and **c** indicate cells at intermediate olfactory epithelium levels with weak immunoreactivity to Pax6 and Gα_0_, probably representing early maturing ORNs. *Open arrows* in **c** point to basal cells with weak immunoreactivity to Pax6 and PCNA, probably early postmitotic cells; *short arrows* in **c** indicate PCNA-negative basal cells with intense immunoreactivity to Pax6 and DCX, possible immature/differentiating ORNs. **d**, **e** Sections across olfactory axons to show the scarcity of Pax6-ir cells along the olfactory nerve during stage 32 (**d**), and their absence in stage 33 (**e**). *Asterisks* indicate the apical surface of the olfactory epithelium; see list for abbreviations. *Scale bars* 100 μm (**a**); 75 μm (**b**); 40 μm (**c**); 300 μm (**d**, **e**)

From stage 32 onwards, the olfactory epithelium became clearly stratified and strongly Pax6-ir cells became progressively restricted to the basal part of the neuroepithelium (Fig. 5a–c), although weakly Pax6-ir cells were also observed at basal and intermediate levels. In the basal olfactory epithelium, weak Pax6-ir cells showed low levels of PCNA immunoreactivity (open arrows in Fig. 5c) and probably correspond to early postmitotic cells, while intense Pax6-ir cells were PCNA negative, but DCX immunoreactive (short arrows in Fig. 5b, c) and they could represent immature/differentiating ORNs. Weak Pax6-ir cells were also observed at intermediate levels of the epithelium (arrowheads in Fig. 5b, c). By their morphology (roundish cells with prominent nuclei), position (middle third of the epithelium), and the weak levels of the Gα_0_-protein (arrowheads in Fig. 5b), they could correspond to maturing ORNs. However, Pax6 expression was absent from fully mature ORNs that were identified by neurobiotin labeling after application of the tracer to the olfactory bulb (Fig. 5a) or by their intense immunoreactivity to the Gα_0_-protein (large arrows in Fig. 5b). It is noteworthy that, despite the increase in density of Pax6-ir cells in the olfactory epithelium throughout this period, the density of Pax6-ir cells along the olfactory nerve and in the vicinity of the olfactory bulb became progressively reduced (Fig. 5d, e).

## Discussion

This study describes for the first time in cartilaginous fish the development of the olfactory nerve and characterizes the nature of some cells observed in the developing olfactory epithelium and associated with the olfactory nerve, particularly, the intriguing Pax6-expressing population that was recently observed along the olfactory pathway during the development of *S. canicula* (Ferreiro-Galve et al. 2012a). Our results also provide information about the main events that take place during the development of the olfactory system in vertebrates.

### Main events of the development of the peripheral olfactory system

The slow development of the lesser-spotted dogfish and the relative large size of embryonic structures were particularly advantageous to analyze developmental processes in the peripheral components of the olfactory system, namely the olfactory epithelium and olfactory nerve. This led us to establish three morphogenetic periods characterized by the appearance of pioneer neurons along the olfactory pathway (pioneer period), the massive migration of cells along the olfactory fibers (migratory period), and the acquisition of the mature organization (maturing period) (see Table 2). These periods constitute a useful framework for comparative research on the development of the olfactory system as the main events considered can be reliably recognized in different groups of vertebrates (mouse: Wray et al. 1989; Mori 1993; Schwanzel-Fukuda and Pfaff 1989; Schwanzel-Fukuda 1999; Balmer and LaMantia 2005; Ikeda et al. 2007; Treloar et al. 2009; Gokoffski et al. 2010; Miller et al. 2010; Blanchart et al. 2011; García-González and de Castro 2011; rat: Valverde et al. 1993; De Carlos et al. 1996; chick: Norgren et al. 1992; Drapkin and Silverman 1999; Fornaro et al. 2001; Maier et al. 2011; zebrafish: Whitlock and Westerfield 1998; Whitlock 2004; Yanicostas et al. 2009). Similarities or novelties observed with respect to other vertebrates may serve as a cue to understand the development and evolution of the olfactory system.

The early events of the olfactory system development include the formation of the olfactory placode and its invagination to form the olfactory pit and the centralward growing of olfactory axons to form the olfactory nerve (see Table 2). Important neurogenic processes take place during these early events, such as the differentiation of the first neurons in the placodal epithelium and the growth of first olfactory axons toward the forebrain, which eventually will join in fascicles to form the olfactory nerve. First evidence of neuronal differentiation in the dogfish olfactory placode was seen at stages 20–21, when the earliest neurons appeared to delaminate from the placode and enter the mesenchyme extending short processes prior to the onset of other migratory events or the establishment of the olfactory nerve. We have identified these early cells as pioneer neurons of the olfactory pathway. Similar precocious neuronal populations have been reported to play a pioneer role in other species (zebrafish: Whitlock and Westerfield 1998; chick: Fornaro et al. 2001; human: Bystron et al. 2006; mouse: Ikeda et al. 2007), where they appear involved in axon guidance from the olfactory placode to the olfactory bulb region of the telencephalon (Whitlock and Westerfield 1998). It is interesting to note that in dogfish embryos at stage 24, shortly after the appearance of pioneer neurons, early postmitotic neurons located within the olfactory epithelium extended their axons (DCX-ir) to contact with the telencephalon, which represent the earliest evidence of olfactory projections. These first contacts between fibers arising from the neuroepithelium and the telencephalon have been considered by other authors as a landmark for the morphological identification of the developing olfactory nerve (Drapkin and Silverman 1999; Maier et al. 2011). As in *S. canicula*, the emergence of olfactory axons from the olfactory pit in rat and chick also appeared delayed with respect to early neuronal differentiation (De Carlos et al. 1996; Drapkin and Silverman 1999).

During the second period, the olfactory nerve of *S. canicula* appeared to be highly cellular, as migrating neurons (DCX and HuC/D immunoreactive) were observed along olfactory fibers. As the density of these cells progressively increased, achieving its maximum at stages 29–30 (the end of the second period) and decreased until they disappear at late stage 32, when the basic mature morphology of the olfactory system is established, the second period has been characterized by the massive neuronal migrations along the olfactory nerve. This second wave of neurons migrating outside the olfactory epithelium was also described in other vertebrates and has been commonly referred as the migratory mass. The most frequently described cells in this migratory mass in a variety of vertebrates include olfactory ensheathing cells (OECs), neurons expressing olfactory receptor genes and the olfactory marker protein (OMP neurons) and LHRH/GnRH positive neurons (see “Introduction”). Nevertheless, there is no strict definition for this term and some authors include pioneer neurons in this concept (Miller et al. 2010). We showed that pioneer neurons observed in *S. canicula* during the first period, before the outgrowth of olfactory fibers from the epithelium, are phenotypically distinct from the population of migrating cells observed along developing olfactory axons during the second and third developmental periods, as only these cells show Pax6 immunoreactivity (see below). Accordingly, we will use the term migratory mass to refer only to the later.

A crucial observation of this study refers to the GFAP-ir population surrounding olfactory axons that was observed in *S. canicula* as early as at stage 29, and which we have identified as OECs. As indicated above, the migratory mass in mammals include OECs that can be readily identified as they express GFAP and wrap bundles of axons (Barber and Lindsay 1982; Smithson and Kawaja 2009; Pellitteri et al. 2010; Forni et al. 2011; Higginson and Barnett 2011). In *Xenopus*, GFAP immunoreactivity, likely expressed by ensheathing glia, has been described along the olfactory nerve from tadpoles to adults (Huang et al. 2005) although the relation between these cells and the olfactory nerve was not cleared by the authors. In teleosts, some markers for mammalian olfactory ensheathing cells also stain the olfactory pathway (Lazzari et al. 2012), but whether a glial ensheathing structure is present in the olfactory nerve of bony fishes is not well known. Present results first reporting cells with immunohistochemical and structural characteristics of OECs in a cartilaginous fish indicate that ensheathing glia is already present in the most ancient radiation of jawed vertebrates.

It has been suggested that bundling of fibers is a property of glial cells (Drapkin and Silverman 1999) and that OECs provide essential growth and guidance cues for ORNs axons (Forni et al. 2011). However, in *S. canicula*, the migratory mass and several olfactory projections bundled together into a single nerve before the ensheathing glia can be detected by means of GFAP immunohistochemistry. Nerve bundling also precedes the expression of glial markers in mouse (Miller et al. 2010) and chick (Drapkin and Silverman 1999). The possibility that immature glia could be present along the olfactory nerve before it expresses GFAP has been considered (Drapkin and Silverman 1999), which is consistent with the finding that the migratory mass in mouse contain undifferentiated cells that are a source of OECs (Blanchart et al. 2011). To our knowledge, whether the immature glia actually guides axons toward the olfactory bulb has not been addressed so far. Moreover, the possibility that other cell types contribute to the signaling required for the establishment and guidance of the olfactory pathway before the maturation of the ensheathing glia cannot be ruled out (see below).

### Pax6 in the olfactory system: characterization and possible roles

Studies in mammals have demonstrated that the expression of *Pax6* gene is indispensable for the normal formation of the olfactory placode, differentiation of the olfactory epithelium and development of the olfactory bulb (reviewed in Nomura et al. 2007). These roles are not exclusive of mammals as the expression of this gene has been demonstrated in components of the developing olfactory system of a basal vertebrate, the shark *S. canicula* (Ferreiro-Galve et al. 2012a). Interestingly that study presented the first evidence in vertebrates of Pax6-expressing cells located along the course of the olfactory nerve, but the nature of such cells was not determined. In the present study, we demonstrate the neuronal nature of Pax6-expressing cells present in different components of the peripheral olfactory system, such as the olfactory placode and epithelium, and along the olfactory nerve. Moreover, we show the existence of different subpopulations of Pax6 cells, which could be playing different roles during development.

#### Pax6 in the olfactory placode and epithelium during development

Different levels of Pax6 expression were detected by means of immunohistochemistry, ranging from a weak and diffuse expression observed at very early stages in cells of the olfactory placode, to a very strong expression that clearly delimitated the nuclei of olfactory epithelium cells from the second developmental period onwards. The relative levels of Pax6 protein in a cell have been correlated to differential functions in the regulation of proliferation and differentiation processes (Hsieh and Yang 2009; Sansom et al. 2009; Ferreiro-Galve et al. 2012b).

In this study, we show that during the first period (early placode formation), weak Pax6 expression extends throughout the highly proliferating olfactory placode, but it is excluded from the neurogenic region, which contains the first postmitotic (though immature) neurons. This fact suggests a possible implication of Pax6 in maintaining the proliferative state of most placodal cells and supports the assumption that it is necessary to maintain low Pax6 protein levels for S phase re-entry, whereas either rapid accumulation or complete downregulation of Pax6 protein levels during the G2/M phase may be required to specify diverse neuronal fates (Hsieh and Yang 2009).

**Fig. 6:**
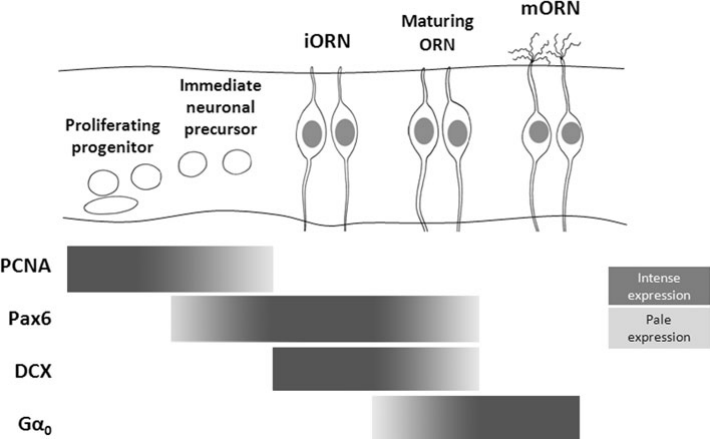
Sequence of markers expressed during the maturation of the ORN lineage. A model relating the sequence of maturation of the ORN lineage with the changes observed along the development in the intensity of labeling of the markers used

During the second period, intense and nuclear Pax6-immunoreactivity was observed first in a subset of postmitotic neurons in the olfactory epithelium, and then it extended from medial to distal parts following the spatio-temporal expansion of neurogenesis, which was visualized by means of neuronal postmitotic markers as HuC/D and DCX. However, not all postmitotic cells in the olfactory epithelium were Pax6-ir. In the chick retina, Hsieh and Yang (2009) showed that, upon the influence of unidentified cues, subsets of progenitors alter their Pax6 level in preparation for cell cycle exit, so that postmitotic neurons achieve and maintain distinct levels of Pax6, ranging from Pax6 negative (in specific cell types) to strongly Pax6-ir (in others). We thus performed double- and triple-labeling assays to determine whether Pax6 expression was related to particular (neuronal or non-neuronal) cell types within the mature epithelium. The mature olfactory epithelium of *S. canicula* has already been described by Ferrando et al. (2010) and consists of three main cell types: basal cells, including globose-like and horizontal-like progenitor cells involved in neuroepithelial renewal and located at the base of the epithelium; sensory cells, including numerous ORNs and quite rare crypt neurons (CNs), located at the middle and upper third of the olfactory epithelium, respectively; and sustentacular cells supporting both epithelial and glial functions, also localized in the upper third. A further cell type has been described in the olfactory epithelium of adult *S. canicula*, the light stained cells (LSCs) for which a role in ionic regulation has been suggested (revised in Ferrando et al. 2006, 2010). In this study, we have characterized the transition from proliferating progenitors to mature ORNs in the postnatal olfactory epithelium attending to the sequential expression of different markers (see Fig. 6). Numerous intense PCNA-ir cells were found in the basal part of the epithelium, though scattered weak PCNA-ir cells were also found within the middle and the apical parts of the epithelium. However, PCNA was absent from cells with round clear nucleus localized in the middle zone of the olfactory epithelium, which can be identified as maturing ORNs in the basis of their morphology. These results are consistent with that reported in the adult olfactory epithelium of *S. canicula* (Ferrando et al. 2010), where PCNA has been found in basal cells (probably globose-like basal cells) and sustentacular (non-neuron) cells, but not in ORNs. We found that weakly Pax6-ir basal cells were also weakly PCNA-ir, which supports the idea that keeping low Pax6 protein levels is necessary for maintaining the proliferative state of cells (Hsieh and Yang 2009). Pax6 has also been described in basal progenitor cells of the olfactory epithelium of the adult mouse (Guo et al. 2010). On the other hand, intense Pax6-ir cells in *S. canicula* were mainly located at basal levels, where they co-distribute, but not colocalize with PCNA-ir progenitor cells, which again supports the hypothesis that rapid accumulation of Pax6 protein levels may be required to specify diverse neuronal fates (Hsieh and Yang 2009). Most of these postmitotic Pax6-ir cells were identified as immature neurons as they also expressed DCX, which has been used as a marker of migrating immature neurons in the central nervous system (Brown et al. 2003) as well as of immature ORNs in the olfactory epithelium of mouse (Murdoch and Roskams 2007). In addition, weak Pax6-ir cells were observed in the middle part of the epithelium. These cells co-expressed low levels of DCX, but additionally showed weak levels of the Gα_0_-protein, which has been largely used as a marker for ORNs (Ferrando et al. 2009). We thus identified these weak Pax6-ir cells as maturing ORNs because of their morphology (roundish cells with prominent nuclei; Ferrando et al. 2007a, b), location (middle third of the epithelium) and the presence of low levels of Gα_0_-protein in their apical processes. Finally, Pax6 expression was absent from fully mature ORNs, which lack DCX and show intense immunoreactivity to protein Gα_0_, as reported in adults (Ferrando et al. 2009). These results strongly suggest that Pax6 is expressed in postmitotic maturing cells belonging to the ORN lineage. In fact, studies in birds demonstrated that olfactory precursors are capable of expressing Pax6 (and *Dlx3*) even when grafted to the trunk and that some of these graft-derived cells eventually differentiate as Hu-positive neurons (Bhattacharyya and Bronner-Fraser 2008). In mouse, Pax6 expression was mainly described in basal progenitors and apical non-neuronal sustentacular cells (Davis and Reed 1996; Behrens et al. 2000), although recent works showed an additional Pax6 expression in a reduced number of neuron-committed Mash1-expressing cells (Gokoffski et al. 2010; Guo et al. 2010). These observations differ from that observed in *S. canicula* (Ferreiro-Galve et al. 2012a and present results), in which Pax6 was strikingly expressed in high numbers of cells of the ORN lineage throughout the olfactory epithelium. Whether this difference could be related to different rates of ORN renewal between species remains elusive. Further studies in other vertebrate groups will be needed to clarify this issue.

#### Pax6 in the migratory mass

Although Pax6 expression was never detected in pioneer neurons of the olfactory pathway (present results), an intense Pax6 expression was observed in a subset of neurons within the migratory mass, which further supports the idea that the pioneer cells and the migratory mass represent different cell populations. To elucidate the identity of the Pax6-positive cells of the migratory mass, we analyzed their spatiotemporal pattern of expression as they migrate from the olfactory epithelium to the olfactory bulb. Although Pax6-ir cells appeared to delaminate from the epithelium, a neural crest origin cannot be discarded. Observations from transgenic mice and cell tracing studies in chick revealed the presence of neural crest-derived cells in the olfactory epithelium, which were able to give rise to all cell types found in the olfactory epithelium and presented morphologic and antigenic properties identical to placode-derived cells (Katoh et al. 2011). In the dogfish migratory mass, Pax6-ir cells were observed in apposition to olfactory axons and they accumulated near the olfactory nerve–olfactory bulb junction (stages 29–31) at the point where the terminal nerve migrating cells appeared to branch off (unpublished observations). As indicated above, different cell types, including OECs, OMP-expressing cells and terminal nerve cells are known to migrate along olfactory fibers during development as part of the migratory mass. Pax6-ir cells have not been described so far within the migratory mass of vertebrates, so we aimed to know whether these cells could belong to any of the cell populations described in the literature. In this work, we proved that Pax6 cells did not colocalize with the glial marker GFAP and expressed the neuronal marker HuC/D. Accordingly, we dismissed their glial nature and ruled out the possibility that they represent OECs. We consider very unlikely that Pax6-ir migrating cells were terminal nerve cells, as we did not detect any Pax6-positive cell in the clusters of branched neurons heading for anterior telencephalon that we identified as belonging to the developing terminal nerve system (unpublished observations). Instead, these cells seemed to head for the vicinity of the olfactory bulb. However, Pax6-expressing cells in the olfactory bulb of *S. canicula* are scarce and mostly restricted to the granular layer (Ferreiro-Galve et al. 2012a). Pax6-ir cells were not detected between the entrance of the olfactory nerve and the olfactory bulb granule layer, which strongly suggests that these cells did not migrate into the granule layer and supports the assumption that Pax6-ir granule cells originate in the telencephalic hemispheres (Franco et al. 2001; Kohwi et al. 2005). Although downregulation of Pax6 expression at the entrance of the olfactory bulb cannot be discarded, the presence of numerous apoptotic cells in the same region (present results) suggests that Pax6 cells could undergone programmed cell death at this point. However, colocalization experiments with apoptotic markers will be needed to confirm this assumption.

Although Pax6 cells have not been described in the migratory mass of other vertebrates, we do not consider that they represent a derived feature of *S. canicula*, or of basal gnathostomes. They do not form part of any specialized structure as those present in the olfactory system of some vertebrates, as the Grueneberg ganglion of mouse (Roppolo et al. 2006) or the accessory olfactory organ of the sea lamprey (Ren et al. 2009). In contrast, Pax6-ir cells are located in regions of the olfactory system that show highly conserved structural and developmental features along evolution, as the olfactory nerve. Therefore, similar cells may have been overlooked in studies of other vertebrates, perhaps because they form a transient population that would be more easily detectable in vertebrates with protracted development and large embryonic brains as *S. canicula*. Moreover, most studies of Pax6 expression are based on the in situ hybridization techniques, which give a lower cell labeling than immunohistochemical methods, although these Pax6 cells have been clearly demonstrated with in situ hybridization (Ferreiro-Galve et al. 2012a). Other possibility is that equivalent migrating neurons are present in the migratory mass of other vertebrates, but they express other gene/s that play a similar role than *Pax6* in the shark. Possible candidates are *Dlx3* and *Dlx5*, which, together with Pax6 appear to act as molecular guideposts for olfactory placode precursors at different developmental stages in the chick (Bhattacharyya and Bronner-Fraser 2008). Various studies have proposed a guidance role for some migrating cells associated with olfactory nerve fibers. In mouse, extraepithelial cells expressing a particular olfactory receptor gene have been described closely associated with outgrowing axons of maturing ORNs that express the same receptor type (Conzelmann et al. 2002). It has been suggested the possibility that the extraepithelial cells might be acting as guideposts or transient targets for exactly those outgrowing axons equipped with the same olfactory receptor type (Conzelmann et al. 2002). These authors were not able to follow the fate of this population as it was no longer detectable after a certain developmental stage. In rat, OMP-expressing cells located along the course of the olfactory axons seemed to disappear during development (Valverde et al. 1993). These cells may represent a group of intervening neurons between the ORNs and the olfactory bulb that serve as hints for olfactory axons to reach their targets. De Carlos et al. (1996) also described cells that detached from the olfactory epithelium and navigated along the course of olfactory fibers, which they identified as the OMP-ir neurons described by Valverde et al. (1993). Several lines of evidence suggest that the Pax6 migrating neurons observed in shark may have a role in the guidance of outgrowing olfactory axons. First, Pax6-ir cells in the migratory mass do not contact the epithelium as they were not labeled after application of neurobiotin in the olfactory epithelium, but rather they were apposed to axons that emerge from Pax6-positive cells anchored to the olfactory epithelium, what highly reminds the scenario observed by Conzelmann et al. (2002). The fact that Pax6 in the olfactory epithelium was specifically expressed by neurons committed to the ORN lineage strongly support this hypothesis. Studies with early markers of ORNs might provide direct evidence that Pax6-ir migrating cells within the shark migratory mass correspond to the ORN-expressing cells described by others. Second, the number of Pax6-ir cells decreases in the olfactory nerve coinciding with the maturation of the olfactory ensheathing glia, whose role in assistance to growing olfactory axons is largely accepted. The possibility that mature OECs could substitute Pax6-expressing cells in their guidance role as development proceeds, cannot be ruled out. In addition, the scarce number of Pax6-ir cells observed at the entrance of the olfactory bulb was found to form corridors between arriving olfactory axons, which further suggests a signaling role for these neurons. Finally, many transcription factors have dual roles in both forebrain morphogenesis and development of axonal pathways (Pratt and Price 2006). Indeed, Pax6 seems to regulate genes implicated in axon guidance including *Sema5A* and *Sema3C* during development of various brain regions (Jones et al. 2002). There is also strong evidence that Pax6 regulates cell–cell adhesion during brain morphogenesis, which is probably important in mechanisms of axon guidance (Pratt and Price 2006). Nonetheless, further functional experiments will be needed to ascertain the precise role of Pax6-ir neurons along outgrowing axons of the shark olfactory pathway.

## Conclusions

The three periods identified by the main events during development of the olfactory system in the shark *S. canicula* constitute a useful framework for vertebrate comparative studies. The present results reveal that processes described as key events during the early development of the olfactory system in amniotes, such as the migration of pioneering neurons along the olfactory nerve, and the existence of a second wave of transient migratory cells (the migratory mass) along the developing olfactory nerve, appeared very early during the evolution of vertebrates. The evidence presented here about the existence of olfactory ensheathing cells of glial nature revealed a common general pattern in the development of the olfactory system of vertebrates, enhancing the importance of cartilaginous fish for understanding the early evolution of this system. Although studies in other vertebrate species are needed before to know if the presence of migrating Pax6 neurons along the developing olfactory nerve form part of the common general pattern, we propose they play a role as guidepost cells for the outgrowing axons of Pax6 olfactory receptor neurons anchored to the olfactory epithelium.

## Abbreviations

iORN: Immature olfactory receptor neuron
mORN: Mature olfactory receptor neuron
Mes: Mesenchyme
OB: Olfactory bulb
OE: Olfactory epithelium
ON: Olfactory nerve
OP: Olfactory placode
OPit: Olfactory pit
ORN: Olfactory receptor neuron
pOB: Primordial olfactory bulb
pOE: Primordial olfactory epithelium
Protoglom: Protoglomerular fields
st: Stage
Tel: Telencephalon
TN: Terminal nerve
v: Telencephalic ventricle
